# Case of a Wound Penetrating the Abdomen and Thorax

**Published:** 1803-09-01

**Authors:** M. Barthe

**Affiliations:** Professor in that University


**Case of a Wound penetrating the Abdomen and Thorax; read at the Medical Society of Montpelier, by M. Barthe, Professor in that University.**

A Boy received, 25 March, a wound with a long cook's knife, and which had been thrown at him by one of his comrades, at the distance of eight steps; it penetrated the left side, between the sixth and seventh ribs, three fingers breadth from this cartilaginous extremity, and which went half way, nearly in a perpendicular direction. The boy remained for some seconds in the position in which he had received it, that is, standing; and seeing himself abandoned by his comrades, who were affrighted at the accident, he determined to withdraw himself the knife. The author saw the young man soon after, together with M. Combes, and were astonished to find the exterior of the wound covered by a fatty mass, which, on examination, proved to be the omentum. The external opening was not more than four or five lines in extent, and the omentum which protruded was in a state of strangulation. M. Combes determined to extend the opening for the purpose of freeing the omentum, but which resisted his efforts to reduce it; he confined himself, therefore, to making a simple dressing. The wound was obviously extremely complicated, as it penetrated the two cavities; yet as the instrument had passed through the muscular portion of the diaphragm, the degree of danger was in some measure lessened. It was agreed immediately to bleed him, and confine him to a tisanne, made of barley water and syrup of violets. The day passed tolerably calm, but towards evening the patient complained of a sense of pain in the epigastric region, extending to the left hypochondrium, with some difficulty in respiration; and although bled a second time, the patient continued much agitated all night, and in the morning the pains already described were much increased, as well as the difficulty of breathing. It was agreed to bleed a third time, after which these symptoms diminished considerably, but increased towards evening, which induced us to recur to a fourth bleeding; the night was passed somewhat easier than the preceding. The third Prof. Pantignon, was called in, who agreed with the author and M. Combes on the nature of the wound. It was decided, that no effort should be made to return the omentum, which, though it were practicable, was not desirable, as it would pass into the cavity of the chest; since it appeared peared most improbable that it could be made to repass through the wounded diaphragm into the abdomen, so that this body remaining in the thorax, would necessarily impede the motion of the lungs; when, on the contrary, by attaching it to the edges of the wound, there were hopes it might adhere to the edges of the pleura and fixed to the cicatrix, that it could not impede the motion of the thoracic viscera. A ligature was, in consequence, thrown round to prevent its return, and the regimen was continued. The evening of this day the patient appearing to suffer much, and to have much difficulty in respiring, he was bled once more. This remedy was indicated as well by the age, by the temperament of the young man, as by the beneficial effects which, at each repetition, were observed to follow. On the fourth day the symptoms were much diminished; no pain, and hardly tension of the epigastric region; heat was less; respiration easy; but the motions of inspiration and expiration succeeded each other with unnatural quickness. The patient was allowed the use of light broth.

On the fifth day suppuration commenced, and increased on the following days, without, however, exceeding the quantity which might be expected from the size of the wound. All the symptoms declined the 6th, 7th, 8th, and 9th days. Nothing particular occurred till the 16th day, when the protruded portion of omentum separated, which was in size equal to a large filbert nut; the patient was allowed greater latitude of diet, and the 30th day he was perfectly well. The author has since learned, that this young man enjoys the most perfect health, and fulfils the duties of a country life, which he has adopted instead of that of a cook, his original profession.—This case appears to us as extraordinary in its nature, as well as in its results. It is obvious that the knife must have penetrated the two cavities, without touching either the apex of the heart, or great extremity of the stomach, both in the neighbourhood of the part where the diaphragm was pierced. It must also have happened, that no artery of any size was divided, as the intercostal, phrenic, or any branch of the gastric; the perpendicular direction in which the knife passed, is the probable cause of these parts having escaped. It appears that the omentum was forced out by expiration. With respect to the result, this case offers the remarkable instance of a ligature of the omentum under these peculiar circumstances. It is not easy to imagine what change took place in that part, pushed through the diaphragm, but but lying within the wound. An isolated fact of this sort, certainly does not destroy what practice has already established on the dangerous nature of wounds of this kind, but affords the truth of this consequence, really philosophical, that, on every point of doctrine, even those best founded and most firmly established, we should admit exceptions from which practical advantages may be sometimes drawn.

